# Multi-centre implementation of an Educational program to improve the Cardiac Arrest diagnostic accuracy of ambulance Telecommunicators and survival outcomes for sudden cardiac arrest victims: the EduCATe study design and methodology

**DOI:** 10.1186/s12873-021-00416-4

**Published:** 2021-03-04

**Authors:** Christian Vaillancourt, Manya Charette, Sarika Naidoo, Monica Taljaard, Matthew Church, Stephanie Hodges, Shannon Leduc, Jim Christenson, Sheldon Cheskes, Katie Dainty, Michael Feldman, Judah Goldstein, John Tallon, Jennie Helmer, Aaron Sibley, Matthew Spidel, Ian Blanchard, Jim Garland, Kathryn Cyr, Jamie Brehaut, Paul Dorian, Colette Lacroix, Sandra Zambon, Venkatesh Thiruganasambandamoorthy

**Affiliations:** 1 Clinical Epidemiology Unit, Ottawa Hospital Research Institute, The Ottawa Hospital, Civic Campus, Rm F649, 1053 Carling Ave., Ottawa, Ontario K1Y 4E9 Canada; 2 Department of Emergency Medicine, University of Ottawa, Ottawa, Canada; 3 School of Epidemiology & Public Health-Faculty of Medicine, University of Ottawa, Ottawa, Canada; 4 Cardiac Arrest Survivor, Study Patient Partner, Toronto, Canada; 5 Central Ambulance Communications Centre, Ottawa Paramedic Service, Ottawa, Canada; 6 Ottawa Paramedic Service, Ottawa, Canada; 7 Department of Emergency Medicine, University of British Columbia, Vancouver, Canada; 8 Provincial Health Services Authority, British Columbia Emergency Health Services, Vancouver, Canada; 9 Center for Health Evaluation and Outcomes Sciences, Providence Health Care Research Institute, Vancouver, Canada; 10 Sunnybrook Centre for Prehospital Medicine, Toronto, Canada; 11 Li Ka Shing Knowledge Institute, St. Michael’s Hospital, Toronto, Canada; 12 Department of Family and Community Medicine, University of Toronto, Toronto, Canada; 13 Department of Research and Innovation, North York General Hospital, Toronto, Canada; 14 Institute of Health Policy Management and Evaluation, University of Toronto, Toronto, Canada; 15 Division of Emergency Medical Services, Dalhousie University, Halifax, Canada; 16 Emergency Health Services Operations, Nova Scotia, Canada; 17 Department of Emergency Medicine, Dalhousie University, Halifax, Canada; 18 Division of Paramedicine, University of Prince Edward Island, Charlottetown, Canada; 19 Island Emergency Medical Services, Prince Edward Island, Charlottetown, Canada; 20 Department of Emergency Medical Services, Alberta Health Services, Calgary, Canada; 21 Department of Community Health Sciences-Cumming School of Medicine, University of Calgary, Calgary, Canada; 22 Alberta Health Services, Edmonton, Canada; 23 Division of Cardiology and Division of Clinical Pharmacology, University of Toronto, Toronto, Canada; 24 International Business Machines (IBM) Canada, Ottawa, Canada; 25 Heart and Stroke Foundation of Canada, Toronto, Canada

**Keywords:** Cardiac arrest, Agonal breathing, Telecommunicators, Cardiopulmonary resuscitation, Resuscitation, Emergency medical services

## Abstract

**Background:**

Sudden cardiac death remains a leading cause of mortality in Canada, resulting in more than 35,000 deaths annually. Most cardiac arrest victims collapse in their own home (85% of the time) and 50% are witnessed by a family member or bystander. Survivors have a quality of life similar to the general population, but the overall survival rate for out-of-hospital cardiac arrest (OHCA) rarely exceeds 8%. Victims are almost four times more likely to survive when receiving bystander CPR, but bystander CPR rates have remained low in Canada over the past decade, not exceeding 15–25% until recently. Telecommunication-assisted CPR instructions have been shown to significantly increase bystander CPR rates, but agonal breathing may be misinterpreted as a sign of life by 9–1-1 callers and telecommunicators, and is responsible for as much as 50% of missed OHCA diagnoses.

We sought to improve the ability and speed with which ambulance telecommunicators can recognize OHCA over the phone, initiate timely CPR instructions, and improve survival.

**Methods:**

In this multi-center national study, we will implement and evaluate an educational program developed for ambulance telecommunicators using a multiple baseline interrupted time-series design. We will compare outcomes 12 months before and after the implementation of a 20-min theory-based educational video addressing barriers to recognition of OHCA while in the presence of agonal breathing. Participating Canadian sites demonstrated prior ability to collect standardized data on OHCA. Data will be collected from eligible 9–1-1 recordings, paramedic documentation and hospital medical records. Eligible cases will include suspected or confirmed OHCA of presumed cardiac origin in patients of any age with attempted resuscitation.

**Discussion:**

The ability of telecommunication-assisted CPR instructions to improve bystander CPR and survival rates for OHCA victims is undeniable. The ability of telecommunicators to recognize OHCA over the phone is unequivocally impeded by relative lack of training on agonal breathing, and reluctance to initiate CPR instructions when in doubt. Our pilot data suggests the potential impact of this project will be to increase absolute OHCA recognition and bystander CPR rates by at least 10%, and absolute out-of-hospital cardiac arrest survival by 5% or more.

**Trial registration:**

Prospectively registered on March 28, 2019 at ClinicalTrials.gov identifier: NCT03894059.

## Background

### Cardiac arrest

Sudden cardiac death remains a leading cause of mortality in Canada, resulting in more than 35,000 deaths annually [1–3]. This means that a cardiac arrest occurs every 12 min [3] and most of those occur in prehospital settings [4]. Most cardiac arrest victims are still active members of society. They collapse in their own home 85% of the time and 50% are witnessed by a family member or bystander [4]. Survivors have a quality of life similar to the general population [5], but the overall survival rate for out-of-hospital cardiac arrest (OHCA) rarely exceeds 8% [4, 6, 7]. Victims are almost four times more likely to survive when receiving bystander CPR, but bystander CPR rates have remained low in Canada over the past decade, not exceeding 15–25% until recently [4, 7].

### Telecommunication-assisted CPR instructions

Telecommunication-assisted CPR instructions, where telecommunicators provide CPR instructions or reminders over the telephone to 9–1-1 callers reporting a victim in cardiac arrest, have been shown to significantly increase bystander CPR rates [8]. However, in the first minutes following cardiac arrest, some victims will take short, labored, noisy, gasping breaths. This abnormal breathing, otherwise known as agonal breathing, may be misinterpreted as a sign of life by 9–1-1 callers and telecommunicators [8–10], and is responsible for as much as 50% of missed OHCA diagnoses [10]. These missed OHCA diagnoses can result in withholding or delaying the delivery of telecommunicator-assisted CPR instructions.

### Prior related work

Over the past 10 years, we have completed a number of unique studies in the field of telecommunication-assisted CPR instructions, leading up to this current project.

1. **Pilot Evaluation of Telecommunication-Assisted CPR Instructions in Ottawa**

This before-after study took place over an 18-month period before and after the introduction of telecommunicator-assisted CPR instructions in the Communication Centre in Ottawa, Ontario. A total of 529 cases were included and bystander CPR rates increased from 16.7 to 26.4% (95% confidence interval (CI) 8.5–11.3%; p-0.006) [8].

2) **Multi-Centre Evaluation of Effectiveness of Telecommunication-Assisted CPR Instructions**

This study took place in several Ontario Communication Centres. In this before-after interrupted time series study including 6494 cases before and after the introduction of telecommunicator-assisted CPR instructions, bystander CPR rates increased from 14.9 to 28.7% (*p* < 0.0001) and survival increased from 4.1 to 5.2% (adjusted odds ratio 1.28; 95% CI 1.02–1.62; *p* = 0.04). Telecommunicators recognized 65.9% of all OHCA. Agonal breathing (present in 22.7% of all OHCA) accounted for 47.6% of missed diagnoses, resulting in inappropriately withheld bystander CPR for 240 victims [11, 12].

3) **Systematic Reviews of Factors Leading to the Recognition of Cardiac Arrest by Telecommunicators**

These two systematic literature reviews were peer-reviewed by the International Liaison Committee on Resuscitation (ILCOR) and provided the scientific background from which the 2010 and 2015 resuscitation guidelines for telecommunication-assisted CPR instructions were derived [13, 14]. The reviews conclude that telecommunicators should receive specific instructions on how to recognize the presence of agonal breathing in order to improve their recognition of cardiac arrest.

4) **National Survey of Factors Associated with the Successful Recognition of Agonal Breathing**

This National Survey was distributed to telecommunicators from 24 cities in four provinces (*n* = 404). The survey was developed using the constructs of the Theory of Planned Behavior, which elicits salient attitudes, social influences and control beliefs suggesting corresponding educational behavior-change techniques to improve recognition of agonal breathing and cardiac arrest [15, 16].

5) **Before-After Controlled Pilot Study**

In this recently-completed study we demonstrated we could improve absolute bystander CPR rates by 12.9% and survival to hospital discharge by 8.4% in Ottawa after implementing a theory-based (TPB) educational program on agonal breathing for telecommunicators. Agonal breathing was present in as many as 25.6% of cases, but was responsible for only 15.8% of missed cases (compared to almost 50% previously) [7].

6) **National Survey of Canadian Communication Centres**

Finally, most recently, a National Survey of all Canadian Communication Centres was undertaken to obtain information on their organizational structure, dispatch systems in use, education curriculum and performance monitoring. The information obtained indicates that most centres utilize the Medical Priority Dispatch System, require a High School diploma as minimum entry level education and telecommunicators receive no or minimal education in recognizing agonal breathing as a sign of OHCA [17].

### Canadian resuscitation outcomes consortium (CanROC)

The Canadian Resuscitation Outcomes Consortium (CanROC) collects population-based data on out-of-hospital cardiac arrest in a registry that aims to capture 100% of OHCA patients assessed by emergency medical services within each contributing site’s catchment area. This initiative builds on the expertise acquired by the three Canadian sites that contributed to the North American Resuscitation Outcomes Consortium (ROC) between the years of 2006 to 2016 [18]. Standardized information on Canadian cardiac arrest victims were collected from Communication Centre records, fire reports, paramedic documentation, defibrillator files and hospital records by British Columbia, Toronto communities (Rescu) and the Ottawa/OPALS Group (Ottawa, Windsor, London, Niagara, Sudbury, Thunder Bay). New funding has allowed these 3 sites to continue registry data collection while also expanding to include other sites across Canada, in an effort to establish a National Cardiac Arrest Registry. This will allow for unique opportunities to obtain current information on cardiac arrest occurrence and prognosis, to study different models of care and delivery, to collaborate on multi-province initiatives and to engage and mentor new researchers. This study will rely on data collected by CanROC to collaboratively obtain key study outcomes quickly and economically.

### Importance of this research

The ability of telecommunication-assisted CPR instructions to improve bystander CPR and survival rates for OHCA victims is undeniable. It is also unequivocal that the ability of telecommunicators to recognize OHCA over the phone is impeded by their relative lack of training regarding the significance of agonal breathing as a symptom of OHCA, and their reluctance to initiate CPR instructions when in doubt. Agonal breathing is present in approximately 25% of all OHCA victims. It is responsible for as much as 50% of all unrecognized OHCA, the consequence of which is withheld bystander CPR and most often death.

#### Hypothesis

We hypothesize that the educational program will increase absolute OHCA recognition and bystander CPR rates by at least 10%, and absolute OHCA survival by 5% or more.

#### Objectives

The overall aim of this research project is to improve the ability and speed with which ambulance telecommunicators can recognize OHCA over the phone, initiate timely CPR instructions, and improve survival for OHCA. Specific objectives are to:

1. Measure the impact of telecommunication-assisted CPR instructions on overall survival rates for OHCA following the implementation of an educational program on agonal breathing.
2. Measure the impact of the educational program on overall community bystander CPR rates.
3. Measure the frequency with which telecommunication-assisted CPR instructions are appropriately provided following the educational program.
4. Quantify the frequency and impact of the presence of agonal breathing on OHCA recognition following the implementation of an educational program.
5. Measure if the educational program decreases time delays before OHCA is recognized and telecommunication-assisted CPR instructions are initiated.
6. Measure the impact of the studied community/site on OHCA recognition and OHCA outcomes.
7. Measure the impact of the telecommunication system/platform used on OHCA recognition and OHCA outcomes.
8. Measure the impact of telecommunicators’ training background and current local training on agonal breathing on OHCA recognition and outcomes.
9. Identify high-quality recordings that will be used to derive a cognitive computing (IBM Watson)-assisted algorithm to rapidly recognize the presence of OHCA during conversations between 9 and 1-1 calls and telecommunicators. (Future project objective)

## Methods/design

### Study design

We propose a multiple baseline interrupted time series (ITS) design (using month as a time unit) which compares our stated outcomes over 12 months before and 12 months after the implementation (over a 1-month transition period, with possible inter-site variability based on site size) of the theory-based educational intervention on recognition of agonal breathing. The ITS design is a robust quasi-experimental design which is ideal for our study because: 1) Withholding this potentially life-saving intervention from control groups could possibly be considered unethical by some review boards; 2) Most sites would only participate if ultimately given an opportunity to implement the intervention; and 3) It will account for heterogeneity across sites in their underlying baseline trends and changes in response to the intervention. In addition, this design will give us the ability to explore the effects of our intervention while taking into account possible seasonal variations and secular trends.

Each site will implement the educational program on agonal breathing at their earliest convenience, and enter into a 12-month evaluation period (12 months/time periods after the implementation of the educational program). The evaluation period for all sites could possibly be spread over 18 months given their respective training completion. This will leave us with 6–12 months to complete data collection and analyses.

Data for the 12-month control period preceding the implementation of the educational program (12 months/time periods) will be obtained retrospectively once a site’s evaluation period is determined. Ambulance Communication Centres are already required to keep their telecommunication recordings for at least 5 years, and CanROC will have already prospectively collected all the other required outcome measures throughout.

### Educational intervention

There is a growing body of literature in social sciences supporting the identification of evidence-based competencies required to effectively change behaviour in health care related interventions [19–21]. The Theory of Planned Behaviour (TPB) can be a useful, systematic tool to identify barriers to and facilitators of change, aiding in the design of appropriate forms of intervention [22–28]. The TPB proposes that the strength of an individual’s intention (or motivation) to engage in a behaviour, and the degree of control they feel they have over that behaviour (perceived behavioural control) are the proximal determinants of engaging in that behaviour [29]. The TPB also proposes that intention strength is determined by three variables: attitudes towards the behaviour, subjective norms, and perceived behavioural control. Those theoretically derived determinants of behaviour can be mapped to specific behaviour change techniques [30]. This approach was successful in clinical trials on smoking cessation [21, 31, 32], colorectal cancer screening [33], and physical fitness [34].

The proposed education interventions were developed using behaviour change techniques specifically mapped to address the modifiable factors identified by our national survey of ambulance telecommunicators [15]. These techniques will include:

1. **Information about the significance of agonal breathing:**
  This first module will consist of a 20-min video detailing information about the early clinical signs of cardiac arrest, including agonal breathing. The original 30-min video used in our pilot study was reviewed in collaboration with a committee of telecommunication leaders from participating sites. Its content was shortened by 10 min, references to existing protocols were updated, and a visual representation of agonal breathing was added. Telecommunicators employed by the participating sites will all review this short educational video individually. Site implementation will commence once all regulatory requirements are met for that site and is expected to be staggered over a 6-month period for all sites. Completion of staff training at each site is expected to be completed within 1 month.
2. **Modeling/demonstration of desired behavioural skills:**
  The video will include a number of real interventions involving the description of agonal breathing by 9–1-1 callers. These interactions are judged to be optimal examples to emulate.
3. **Rehearsal of desired skills:**
  A second, shorter module will be updated with new cases of agonal breathing and interactions with 9–1-1 callers and completed once in the middle of the intervention period.
4. **Monitoring/reinforcement and feedback:**
  Each time an ambulance telecommunicator is involved with a cardiac arrest call, they will be provided with a report card which will include various time intervals measured during the review of the recording (time to cardiac arrest recognition, time to initiation of chest compressions), whether or not the victim was indeed in cardiac arrest upon EMS arrival at the scene, and an opportunity to review their performance with their supervisor. We understand that not all sites will have the resources in place for any or all of these feedback measures. We will work with each site to determine their capacity.

### Setting

This study will take place in 10 sites/ambulance Communication Centres distributed in 5 Canadian provinces/regions. They are all associated with sophisticated high-performing paramedic services and receiving hospitals already providing OHCA outcomes data to the CanROC network. Together, these 10 ambulance Communication Centres provide services to an estimated population of 13,919,809 [range 107,406 – 4,800,000], see Table 1. They process more than 1,725,000 9–1-1 calls per year, approximately 8312 of which are for eligible non-traumatic OHCA for which resuscitation is attempted. These sites have also been selected because their ambulance telecommunicators have various training backgrounds, and utilize various types of systems/platforms. All the selected Communication Centres have been providing telecommunication-assisted CPR instructions since or before April, 2004. Our research group has a decade-long history of collaboration with most of these partners. They have all provided a letter in support of this proposed project.

**Table 1:** Annual 9–1-1 call volume, with sudden cardiac death distribution, per study site catchment area

Province	Community	Catchment Population	9–1-1 calls/year	Eligible SCD cases/year
British Columbia	Entire Province	4,800,000	600,000	2800
Alberta	Edmonton	4,100,000	600,000	2400
	Calgary			
Ontario	Toronto	2,790,000	280,000	1650
	London	455,526	66,000	291
	Niagara	431,346	60,000	250
	Sudbury	161,531	14,000	134
	Thunder Bay	107,406	9000	102
Nova Scotia	Entire Province	1,000,000	84,000	585
PEI	Entire Province	146,000	12,000	100
	Total	13,991,809	1,725,000	8312

### Study population

All patients with prehospital cardiac arrest (absence of a detectable pulse, unresponsiveness, and apnea) meeting the following criteria will be enrolled:

1. Presumed cardiac origin;
2. Occur in the catchment area of the participating Communication Centre and corresponding CanROC site;
3. Where resuscitation is attempted by a bystander and/or the emergency responders.

Case definitions will follow the Utstein Style guidelines for reporting cardiac arrest data [35, 36]. The number of patients not in cardiac arrest, for which telecommunication-assisted CPR instructions were implemented by the 9–1-1 caller as a result of the telecommunicators’ intervention, will also be reported.

The following patients will be excluded:

1. Cardiac arrest witnessed by paramedics after their arrival (no opportunity for bystander intervention);
2. Patients who are “obviously dead” as defined by the Ambulance Act (decomposition, rigor mortis, decapitation);
3. Trauma victims, including hanging and burns;
4. Patients with cardiac arrest clearly of other non-cardiac origin including drug overdose, carbon monoxide poisoning, drowning, exsanguination, electrocution, asphyxia, hypoxia related to respiratory disease, cerebrovascular accident and documented terminal illness.

### Ethical considerations

We will seek full Research Ethics Board (REB) approval. Informed consent will not be required because patients are neither being targeted by any experimental intervention nor having their identifiable personal information used for research. Patients will undergo therapy and procedures normally provided either inside or outside the hospital and will not be exposed to undue risk or discomfort. Telecommunication-assisted CPR instructions are provided using a protocol that will not be modified as part of the proposed study. Strict patient confidentiality and safeguarding of all study-related documents will be assured, and the trial is publicly registered.

A note on the safety of providing CPR instructions to rare victims not in cardiac arrest: Providing CPR instructions to a population seemingly in cardiac arrest (unconscious and not breathing, or not breathing normally) is a practice supported by the 2015 International Consensus Guidelines on CPR in that, when in doubt about the presence or absence of signs of life, it is recommended to err on the safe side and initiate CPR [37]. For various reasons, since telecommunication-CPR instructions do not always result in CPR being performed on the victim, we observed in our Ottawa pilot study that a very small number of victims erroneously believed to be in cardiac arrest (4.4%) received bystander CPR [7]. After careful review of de-identified in-hospital medical records for every such patient, our independent patient-safety expert panel concluded that no physical injury could be attributed to the provided CPR. Although rib fractures and sternal fractures have been observed in as many as 30 and 15% of cardiac arrest victims during autopsy [38–41], another group from Seattle also failed to observe any adverse event resulting from CPR provided to patients not in cardiac arrest [42].

### Case identification

A list of potential eligible cases will first be identified by each ambulance Communication Centre for each month of the study period. The calls will be flagged using a combination of procedure and complaint codes for inclusivity. Dispatch platform systems differ between sites, thus, details will be outlined with each Communication Centre to ensure flagging of cases is consistent. The list will be sent to the Study Coordinating Centre in Ottawa for review.

The Coordinating Centre will cross-check each list against the CanROC list of confirmed and eligible cases for that site over the same time period. Cases will be matched using only run number and date and time of call to establish eligibility. The inclusion and exclusion criteria will be applied based on CanROC details to arrive at a monthly list of eligible cases from which data will be extracted.

### Classification of calls

After comparing the ambulance Communication Centre and CanROC lists, each cardiac arrest case will be classified into one of the following groups:

1. **Confirmed, eligible:** Cases that appear on both the Communication Centre list and the CanROC list and meet the study eligibility criteria. Further data will be collected from the 9–1-1 recording and the CanROC Registry.
2. **Confirmed, ineligible:** Cases that appear on both lists, but do not meet all study eligibility criteria. No further data will be collected from these cases.
3. **Missed:** Cases in the CanROC registry that meet the study eligibility criteria but were not flagged by the Communication Centre. The Communication Centre will be contacted to pull these cases, and further data will be collected from the 9–1-1 recordings and the CanROC Registry.
4. **Overcalls:** Cases that are flagged by the Communication Centre as possible OHCA cases but do not appear in the CanROC Registry. We will collect further information from the Communication Centre for these calls.

### Sources of information

Each eligible, missed or overcalled case will be reviewed in detail using a variety of sources:

1. **Communication Centre:** Pre-specified quality assurance reports generated by the dispatch software in use as well as the 9–1-1 audio recordings will be available for the eligible, missed and overcalled cases. These sources will be reviewed to obtain information on key time intervals, caller/bystander characteristics, presence of agonal breathing, and details on telecommunication-assisted CPR.
2. **CanROC Registry:** Each eligible and missed case will be linked to the CanROC Registry by run number and call date to locate standardized data that are being collected by the corresponding CanROC Sites. The CanROC minimal data set includes inputs from a variety of sources including: paramedic (ambulance) call reports, defibrillator files, fire medical assist reports, dispatch times and receiving hospital records.

### Method of assessment and data collection

#### Objective 1 (primary outcome)

Overall survival rate to hospital discharge: will be assessed via hospital medical records or via the coroner’s office among study-eligible patients. The CanROC Study group has been using this strategy successfully for more than a decade.

#### Objective 2 (and following secondary outcomes)

Overall community bystander CPR rate: “Bystander” CPR is performed by a person who is not part of an organized emergency medical system. Physicians, nurses, and paramedics may be described as performing bystander CPR if they are not part of the emergency response system involved in the victim’s resuscitation, but bystander usually refers to the untrained lay public [35]. “CPR” is an attempt to restore spontaneous circulation by performing chest compressions with or without ventilations.

The presence or absence of ongoing bystander CPR will be assessed by the first member of the emergency response team to arrive at scene, and is routinely documented on their patient care record.

#### Objective 3

- Successful completion rate of telecommunication-assisted CPR instructions: is defined as CPR instructions provided by an ambulance telecommunicator to a 9–1-1 caller reporting a cardiac arrest victim, resulting in the administration of chest compressions to that victim by a bystander before EMS arrival. The recordings of all eligible OHCA calls from participating Communication Centres will be reviewed using an existing, standardized, and piloted data collection tool to collect information on variables pertaining to the successful delivery of telecommunication-assisted CPR instructions.
- Count of CPR instructions leading to chest compressions in rare victims not in cardiac arrest: As mentioned previously, providing CPR instructions to a population seemingly in cardiac arrest (unconscious and not breathing, or not breathing normally) is a practice supported by the 2015 International Consensus Guidelines on CPR [37]. After careful review of de-identified in-hospital medical records for every such patient, our independent patient-safety expert panel concluded that no physical injury could be attributed to the provided CPR [7]. For this reason, we do not plan (nor will we have the resources) to repeat the extensive in-hospital evaluation of such cases in this large, pragmatic, multi-centre study. Nonetheless, we plan to use the same standardized-piloted data collection tool as above to report the frequency with which CPR instructions led to chest compressions in rare victims not in cardiac arrest.

#### Objective 4

- Agonal breathing: Can be defined as abnormal breathing, gurgling sound, moaning, or otherwise. Ambulance telecommunicators are required by protocol to inquire about the presence or absence of agonal or abnormal breathing. The standardized-piloted data collection tool will be used to independently document the presence or absence of agonal breathing, as described by the 9–1-1 caller to the telecommunicator. In a previous evaluation of inter-rater reliability for the recognition of agonal breathing, we observed substantial agreement (kappa statistics reported) for the presence of agonal breathing between telecommunicators and investigators, between telecommunicators, and within themselves when asked to repeat the exercise 4 months later [8].
- Recognition of OHCA by telecommunicators: According to protocols, ambulance telecommunicators assume the presence of cardiac arrest when the 9–1-1 caller describes a victim that is lifeless (unresponsive, not breathing, or not breathing normally).
  Recordings of the OHCA cases from participating ambulance Communication Centres will be reviewed and reasons why cardiac arrest may not have been recognized by the telecommunicator (including the presence of agonal breathing) will be documented.
- Determination of cardiac origin of OHCA: We will use the Utstein definition for cardiac arrest of cardiac origin [35]: “If an EMS provider or physician did not witness the cardiac arrest, he/she may be uncertain as to whether a cardiac arrest actually occurred. An arrest is presumed to be of cardiac etiology unless it is known or likely to have been caused by trauma, submersion, drug overdose, asphyxia, exsanguination, or any other non-cardiac cause as best determined by rescuers.”

Cardiac origin of OHCA is usually first suspected/documented by the paramedic crew responding to the scene, and subsequently confirmed using hospital discharge summaries or coroner’s data. This information will be readily available from the CanROC registry.

#### Objective 5

Speed with which OHCA is recognized and telecommunication-assisted CPR instructions are initiated: All 9–1-1 communications are recorded on digital files (for e.g. Canadian Voice Data Switching (CVDS) files) which include a running digital clock. We will record the time at which OHCA was recognized by telecommunicators, the time at which CPR instructions are initiated, and calculate time intervals from the initiation of the 9–1-1 call to these time stamps.

#### Objectives 6, 7, and 8

We are planning pre-determined subgroup analyses to study the impact of: 6) the studied community/site; 7) the telecommunication system/platform used; and 8) the telecommunicators’ training background and current local training on agonal breathing on the previously stated primary and secondary outcomes (Objectives 1–5).

#### Objective 9 (not to be reported as part of project #1 per se)

Collect de-identified high-quality recordings for use in proposed Project#2: All 9–1-1 communications are recorded on digital files (for e.g. Canadian Voice Data Switching (CVDS) files), and are kept for a period of 5 years by ambulance Communication Centres. We intend to archive de-identified copies of this material for a period of 10 years, as required by standard operative procedures.

### Data management and flow

The Data Management and Coordinating Centre for the study is located at the Ottawa Hospital Research Institute (OHRI) in Ottawa, Ontario. The Ottawa Methods Centre, located at the OHRI, will develop, maintain and host a secure, password protected web-based study database into which study personnel with granted access will input study data.

The Data Management and Coordinating Centre for the CanROC Registry is located at St. Michael’s Hospital in Toronto, Ontario and is managed by the Applied Health Research Centre (AHRC). The CanROC Registry is a web-based secure, password-protected data entry interface and corresponding database. In most cases, the CanROC variables will be obtained from the participating local CanROC site. Where direct acquisition is not available, a request will be made for transfer from the CanROC Registry.

**Fig. 1:**
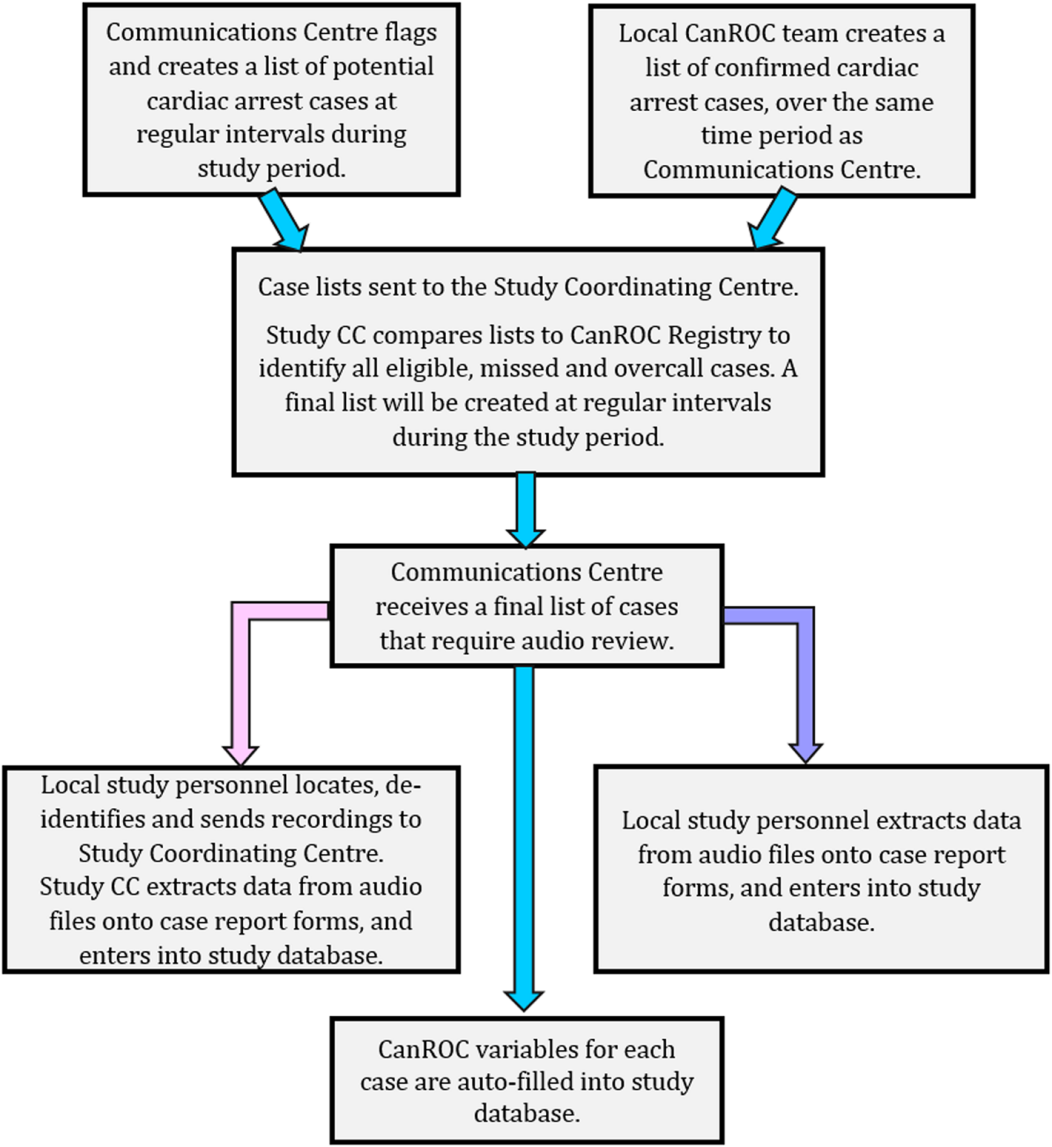
Proposed data flow options

Data flow (Fig. 1) for the study will involve regular interactions between participating Communication Centres, the study Coordinating Centre and the CanROC Site or CanROC Coordinating Centre.

Each month during the study period, a list of potentially eligible cases will be generated by each participating Communication Centre and sent to the Study Coordinating Centre in Ottawa for classification. The Coordinating Centre will obtain a parallel list of study-eligible, confirmed cardiac arrest cases over the same time period, from the corresponding CanROC Site or the CanROC Registry. The two lists will be compared and classified into one of the four categories (eligible, ineligible, missed, overcalled). This final list of eligible cases will then be sent back to the Communication Centre for preparation. Depending on the resources available at each site, one of the following will then occur:

1. Study staff at each Communication Centre will locate the 9–1-1 recordings, remove all identifying information and securely transfer them to the Study Coordinating Centre in Ottawa for review. Trained study staff in Ottawa will extract the study data and enter it into the study database.
2. Study Coordinating Centre will train local study staff to review 9–1-1 recordings, extract the study data and enter it into the study database.

The Study Coordinating Centre will adopt a pragmatic approach to accommodate possible manpower limitations, provincial regulations, privacy concerns or other issues. Where there are deviations from these processes, the revised flow will be documented.

Once the recordings have been reviewed and the required information obtained, the Study Coordinating Centre will request the CanROC variables for the eligible and missed cases.

The data will be pushed securely to the study database and will be linked via run number and call date.

Extensive steps have been taken to ensure the protection of patient privacy. At the Study Coordinating Centre, all incoming data will have been transferred securely and stripped of identifying information. No direct identifiers are included in the data being collected, information which is obtained during routine, standard care with no direct contact with research participants. De-identified electronic files will be stored securely on a network folder with access limited to essential study personnel. Paper files are stored in a locked cabinet, in a locked room.

The CanROC Site data will be entered using a secure, password-protected interface on the CanROC Registry. No direct personal health identifiers are collected in the CanROC Registry and no source documents are uploaded. The transfer of the CanROC variables to the Study Coordinating Centre will be outlined in an agreement agreed upon by all parties and will occur via a secure File Transfer Protocol (FTP).

### Data analysis

#### Overall survival to hospital discharge and community bystander CPR rates – objectives 1 and 2

Individual time series plots for each of the 5 regions will be created to visually inspect the immediate and long-term effects of the intervention, and the presence of trends, cyclical patterns, and outliers. The aggregated monthly rates in each region, expressed as percentages, will be analyzed using a simple linear segmented autoregressive model. To avoid instability due to small numerators and denominators at some sites, multiple sites within the same region will be pooled for the analysis where necessary. The model will include fixed terms for time pre-intervention, intervention, and time post-intervention. The implementation period will be censored from analysis by coding the intervention variable as missing during these months [43]. The model will be estimated using maximum likelihood estimation. Statistical significance will be obtained as the estimated beta coefficients divided by their standard errors with standard errors accounting for the autoregressive parameters. As the primary analysis will be conducted at the aggregate level, no covariates will be adjusted for in this analysis. We will assess serial autocorrelation, non-stationarity, and seasonality using the Durbin-Watson statistic, Dickey-Fuller unit root test, and visual inspection of residual plots against time. The fit of the final model will be assessed by inspecting residuals around the predicted regression lines. In case of substantial non-normality, the analysis will be conducted on the log or logit scale. The effect of the intervention will be expressed for each outcome as intercept and slope changes, where the former can be interpreted as the immediate effect of the intervention and the latter as the gradual effect of the intervention on each outcome over time. We will also evaluate the difference, at 12 months post-implementation, between the fitted post-implementation rates and the projected rates estimated using only the pre-intervention data. This represents the counterfactual effect, that is, the difference between the observed rate and the rate that would have been observed had the intervention not been implemented. We will express these differences on absolute or relative scales, with 95% confidence intervals (CI) calculated using the method of Zhang et al. [44]

To obtain pooled estimates across all five regions, two methods will be used: First, we will use a random effects meta-analytical approach to pool the five region-specific intervention effect estimates and their standard errors. Next, we will use generalized linear mixed effects regression of the site-specific data with random intercepts for the sites and random slopes for time. All analyses will be conducted using SAS version 9.4 (SAS Institute, Inc., Cary, North Carolina).

#### Successful completion of telecommunication-assisted CPR instructions and frequency of CPR instructions leading to chest compressions in rare victims not in cardiac arrest – objective 3

We will use descriptive statistics with 95% CIs to describe successes in various stages of the implementation of CPR instructions (e.g. instructions offered, provided, completed, chest compressions initiated), and the frequency with which chest compressions are (rarely) going to be provided to a victim not in cardiac arrest.

#### Frequency and impact of agonal breathing on OHCA recognition by telecommunicators – objective 4

The frequency and impact of agonal breathing (proportion of missed OHCA as a result of agonal breathing) will be reported using kappa and descriptive statistics with 95% CI. Sensitivity, specificity, negative and positive predictive values will be computed with 95% CIs for the ability of ambulance telecommunicators to recognize OHCA over the telephone. OHCA recognition rates will be compared in the before and after periods using statistical analyses described above for Objectives 1 and 2.

#### Speed with which OHCA is recognized and telecommunication-assisted CPR instructions are initiated – objective 5

Descriptive statistics with 95% CIs will be used to describe various time delays occurring during telecommunication-assisted CPR instructions (e.g. time to OHCA recognition, to initiation, and to completion of instructions). We will compare these time intervals (continuous variable) in the before and after periods using statistical analyses similar to those described above for Objectives 1 and 2.

#### Objectives 6, 7, and 8

In addition to the meta-analytic (across all sites) approach proposed for the analyses of Objectives 1 and 2, we plan to stratify our interrupted time series analyses to explore the impact of 6) the studied community/site; 7) the telecommunication system/platform used; and 8) the telecommunicators’ training background and current local training on agonal breathing on the previously stated primary and secondary outcomes (Objectives 1–5).

### Sample size

Calculating sample sizes for interrupted time series analyses is often done pragmatically. Per recommended methodological standards, the study will include 12 time periods (monthly repeated measures) before and after the introduction of the educational intervention, each time period providing far more than the recommended 50–100 cardiac arrest cases per month [45, 46]. Based on the estimated 6000–7000 cardiac arrest cases available per year (with 12,000–14,000 cases in total in the study), from the proposed regions, there will be an estimated 500 cardiac arrest cases and 50 survivors per month for each of the time units in the before and after time periods.

## Discussion

The proposed research study will improve clinical care and outcomes for cardiac arrest victims, use various knowledge translation strategies to engage potential bystander CPR providers, engage 9–1-1 telecommunication officers in research activities, develop new research centers of excellence among Canadian ambulance Communication Centers, expand CanROC’s research network and capabilities, and create new commercial technology (IBM-Watson collaboration). We estimate absolute bystander CPR and survival rates could increase by 20 and 10% respectively, after the completion of the entire research program. Given the reported incidence of sudden cardiac death in Canada (35,000/year), we could save as many as 3500 victims/year, positively impact their families, decrease health care burden, and return active members of society to a productive life.

## Data Availability

Data produced in the study may be accessible via the corresponding author upon reasonable request and after review by a data access committee.

## Abbreviations

AHRC: Applied Health Research Centre
CanROC: Canadian Resuscitation Outcomes Consortium
CI: Confidence Interval
CPR: Cardiopulmonary Resuscitation
FTP: File Transfer Protocol
ITS: Interrupted Time Series
OHCA: Out-of-hospital Cardiac Arrest
OHRI: Ottawa Hospital Research Institute
ROC: Resuscitation Outcomes Consortium
